# A pan-cancer perspective of matrix metalloproteases (MMP) gene expression profile and their diagnostic/prognostic potential

**DOI:** 10.1186/s12885-019-5768-0

**Published:** 2019-06-14

**Authors:** Emily Gobin, Kayla Bagwell, John Wagner, David Mysona, Sharmila Sandirasegarane, Nathan Smith, Shan Bai, Ashok Sharma, Robert Schleifer, Jin-Xiong She

**Affiliations:** Center for Biotechnology and Genomic Medicine, Medical College of Georgia, Augusta University, Augusta, GA 30912 USA

**Keywords:** Gene expression, MMPs, Matrix metalloproteases, Biomarkers, Survival, Diagnosis, Prognosis, TCGA

## Abstract

**Implication:**

By understanding Matrix Metalloprotease (MMP) dysregulation from a pan-cancer perspective, this study sheds light on the diagnostic potentials of MMPs across multiple neoplasms.

**Background:**

MMPs are intriguing genes related to cancer disease progression, functional promotion of angiogenesis, invasion, metastasis, and avoidance of immune surveillance. Many studies have noted these genes are frequently upregulated in cancer. However, expression patterns of all MMPs and their diagnostic and prognostic potential have not been investigated in a pan-cancer perspective.

**Methods:**

The Cancer Genome Atlas (TCGA) data were used to evaluate diagnostic and prognostic potential of 24 MMPs in fifteen different cancer types. Gene expression measured by RNA-seq was analyzed by differential expression, hierarchical clustering, and ROC analysis for individual genes and in combination.

**Results:**

*MMP1, MMP9*, *MMP10*, *MMP11*, and *MMP13* were almost universally upregulated across all cancers, with significant (*p* < 0.05) fold change (FC > 2) in ten of fifteen cancers. *MMP3*, *MMP7*, *MMP12* and *MMP14*) are significantly up-regulated in at least 10 cancer types. Interestingly, *MMP2*, *MMP7*, *MMP23B*, *MMP27* and *MMP28*) are significantly down-regulated in seven to nine cancer types. Multiple MMPs possess AUC’s > 0.9 in more than one cancer. However, survival analyses suggest that the prognostic value of MMPs is limited to clear cell renal carcinoma.

**Conclusions:**

Most MMPs have consistently increased gene expression across cancers, while several MMPs have consistently decreased expression in several cancer types. Many MMPs have diagnostic value individually or in combination, while the prognostic value of MMPs is restricted to one subtype of kidney cancer.

**Electronic supplementary material:**

The online version of this article (10.1186/s12885-019-5768-0) contains supplementary material, which is available to authorized users.

## Background

MMPs have been extensively studied for nearly 40 years and originally were noted for their role in degrading the extracellular matrix (ECM) [1]. Structurally, MMPs contain similar catalytic inhibitory domains. The catalytic domains add specificity to the target for degradation of each MMP. Traditional classification of MMPs delineates based on the first identified target of degradation. Over the years, MMPs have been found to play a remarkable number of regulatory roles at the cellular level in pathways such as apoptosis, immunity, cellular migration, and angiogenesis [2]. MMP functionality often complements classical tumor properties leading to invasion, immune system avoidance, and metastasis. Given these qualities, MMPs play a major role in carcinogenesis [1–4]. Table 1 provides an overview of specific role of each MMP in cancer.

**Table 1:** MMPs in Cancer

MMP	Role in Cancer
Collagenases	
1	Initial invasion, promotes metastasis [5, 6]
8	
13	Growth, invasion, and angiogenesis of skin squamous cell carcinoma [7]
Matrilysins	
7	Contributes to invasive potential, proliferation, anti-apoptotic, immune surveillance [1, 8]
26	Activates MMP-9 in prostate cancer, role in early skin carcinogenesis [9, 10]
Metalloelastase	
12	Protective inhibition of tumor growth, anti-angiogenic [11]
Stromelysins	
3	Invasion, metastasis, and epithelial to mesenchymal transition [12–14]
10	Invasion, migration, and growth; prevents tumor cell apoptosis; produces angiogenic and metastatic factors [15–17]
11	Produced by peritumoral stromal fibroblasts; regulates early tumor invasion, implantation, and expansion; prevents apoptosis of early cancer cells [18–20]
Gelatinases	
2	Proteolytic degradation of extracellular proteins in tumor invasion, collagenolytic pathway driver for lymphatic vessel formation, tumor angiogenesis [1, 15, 16]
9	Proteolytic degradation of extracellular proteins during tumor invasion [1, 15]
Enamelysin	
20	Synthesized in odontogenic tumors [21]
Membrane-Type	
14	Cleaves other pro MMPs (mainly MMP2) to activate them, role in invasive blood vessel growth, and promoting metastasis. In vitro has been shown to promote invasion [22, 23]
15	In vitro shown to play role in epithelial to mesenchymal transition, promotes angiogenesis [24, 25]
16	In vitro promotes invasion and metastasis [26, 27]
17	Induce angiogenesis promote growth and metastasis [25, 28]
24	Progression in brain tumors, aides in migration and metastasis [29, 30]
25	In vitro tumor growth promoter [31]
Other	
19	In vitro modulates proliferation, adhesion, and metastasis [32, 33]
21	Expression changes associated with cancer prognosis. [34]
23A	Expression levels altered in multiple cancers. Urinary levels decreased in renal cell carcinoma. [35, 36]
23B	
27	
28	Promotes epithelial to mesenchymal transition, promotes invasion and metastasis [37]

Dysregulation of MMP expression has been noted in numerous studies at the protein and RNA levels in many cancer types (Table 1). Frequently, MMP dysregulation associates with prognostic differences, a trend noted in breast, ovarian, and colon cancers [4, 31]. This manuscript uses data from The Cancer Genome Atlas (TCGA) to examine differences in RNA expression of MMPs in a comprehensive manner. By investigating data from fifteen TCGA cancer types, we seek to: 1) identify patterns of MMP dysregulation, 2) associate MMP expression to patient survival in all 15 cancer types, and 3) integrate the TCGA data with previously published data to gain pertinent insight regarding MMP on tumorigenesis and prognosis.

## Methods

### TCGA datasets

The TCGA gene expression (RNAseq) data (IlluminaHiSeq: log2-normalized_count+ 1) was downloaded from Xena browser (https://xenabrowser.net/datapages/). The fifteen cancer types selected had at least ten patients with adjacent normal samples. The cancer types are denoted by their TCGA four-letter abbreviation (See Abbreviations). Statistical analyses were performed to compare and contrast the expression levels of 24 MMP genes.

### Statistical analyses

All statistical analyses were performed using the R language and environment for statistical computing (R version 3.2.2; R Foundation for Statistical Computing; www.r-project.org). The normalized counts were log2 transformed prior to all statistical analyses to achieve a normal distribution. Expression differences between cancer patients and adjacent normals were initially examined using a t-test where the amount and significance of change were depicted using fold change (FC: Cancer vs Adjacent Normal) and *p*-value (pval). Fold change was calculated as the median gene expression level. Trends in expression differences were identified using unsupervised clustering. Diagnostic power of expression differences (Cancer vs. Adjacent Normal) of individual, or combinations of, MMPs was assessed using the area under the curve (AUC) of the receiver operating characteristic (ROC). Sensitivity values were calculated at various specificity thresholds (90%; 95%; 99%; 100%). Cox proportional hazards models were used to evaluate the association of gene expression levels on overall survival (diagnosis to date of death). Survival data was obtained from the TCGA patient phenotype files. Patients who at the time of analysis were alive with no evidence of disease were censored at the date of last follow-up visit. Kaplan-Meier survival analysis and the log-rank test were used to compare survival differences in groups separated by median expression level at multiple thresholds. Each cancer was considered individually. Multivariate analysis was performed to identify combinations of proteins that had significant findings in the univariate analysis. Data was visualized using R software and Tableau 10.4 (Tableau Software, www.tableau.com).

## Results

### MMP expression difference between Cancer and Normal

**Fig. 1:**
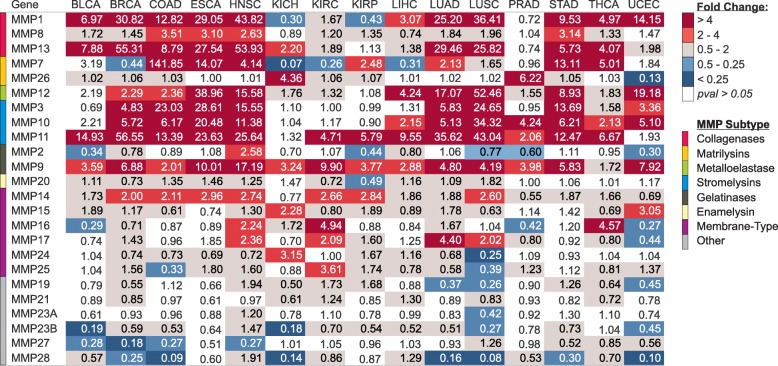
Differential gene expression of 24 matrix metalloproteinases (MMPs) in 15 different cancer types. Fold change and *p*-values shown were obtained through comparison of unmatched control tissue (N between 11 and 114) to tumor tissue (N between 66 and 1097). Fold change was calculated as the median expression of a gene in tumor divided by the median gene expression in adjacent normal tissue

Gene expression differences for the twenty-four MMP family members were analyzed in fifteen different cancer types in the Cancer Genome Atlas (TCGA) (Fig. 1). The most prevalent gene expression changes were upregulation as opposed to downregulation in tumor tissue versus control tissue. MMP subtypes had similarities in expression patterns, with dramatic upregulation in cancer for the majority of collagenases, matrilysins, metalloelastase, and stromelysins. Other MMPs that showed significant differences were strong upregulation of *MMP14* and downregulation of *MMP23B*, *MMP27*, and *MMP28*. The most significant dysregulations were among collagenases and stromelysins. *MMP11*, a stromelysin, was significantly upregulated in all cancer types except KICH and UCEC (*p* = 1E-07 to 1E-122). The other stromelysins, *MMP3* and *MMP10*, were significantly upregulated in 7 cancer types and 10 cancer types respectively. *MMP9*, a gelatinase, was significantly upregulated in twelve of the fifteen analyzed cancer types (p = 1E-05 to 4E-27). Collagenases, *MMP1* and *MMP13*, were significantly altered in 11 cancer types and 12 cancer types, respectively (*p* = 5E-05 to 1E-77).

Across cancer types, lung squamous (LUSC) and uterine corpus endometrial (UCEC) were distinct from the other cancer types with several significantly downregulated MMPs (Fig. 1). Additionally, renal cancers comparatively lacked significant upregulation of MMPs compared to adjacent normal tissue. This pattern is most noticeable among collagenases and stromelysins other than *MMP11*.

### MMP expression is heterogeneous across cancer types

**Fig. 2:**
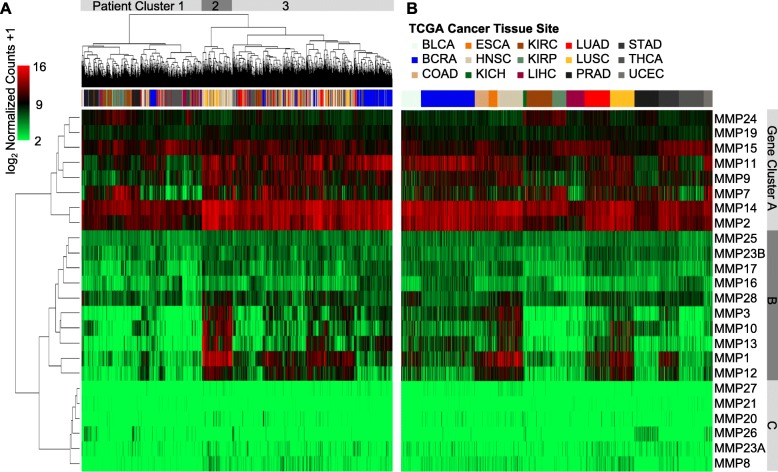
Heat map representing color coded expression levels of 24 differentially expressed MMP genes in 15 different cancer types. Gene expression values are colored from green (low expression) to red (high expression). **a**: Patient expression data hierarchically clustered **b**: Patient expression data grouped by cancer type. Clusters of patients and genes are labelled

Expression of MMPs has a large degree of heterogeneity across cancers (Fig. 2). Some MMPs had very high numbers of transcripts present (gene cluster A), while six MMPs in gene cluster C (*MMP27*, *MMP21*, *MMP20*, *MMP26*, *MMP23A*, and *MMP8*) featured scant numbers of transcript copies across any cancer tissue (Fig. 2a and b). The cancer types with the most significant changes in MMP gene expression were LUSC and HNSC (19 and 15 MMPs, respectively), and patients from these cancers generally clustered together upon unsupervised hierarchical clustering as seen in cluster 2 of Fig. 2a. This grouping in cluster 2 features high expression of gene cluster B genes (*MMP3*, *MMP10*, *MMP13*, *MMP1*, and *MMP12*) in a pattern that is relatively unique among cancer types.

Among the high abundance MMPs, *MMP9*, *MMP11*, *MMP14*, and *MMP2* had marked differences in expression among the three patient clusters. These genes were extremely abundant in breast, esophageal, head & neck, lung, and stomach cancers compared to the other tumor types.

### MMP11 and MMP13 are nearly universally upregulated in cancer

**Fig. 3:**
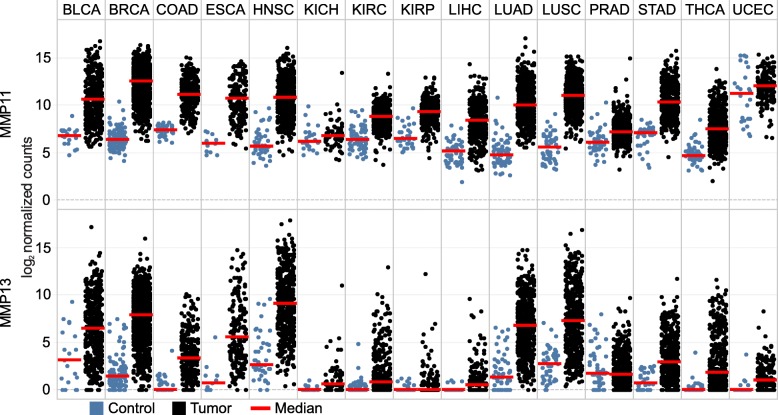
Gene expression of MMP11 and MMP13 across 15 TCGA cancer types. Expression values represented are the normalized counts represented in a log_2_ scale. Thus, a difference of one represents a two-fold expression difference. Medians (red bar) for adjacent normal (blue) and cancer (black) tissues are shown

*MMP11* and *MMP13* expression was dramatically higher in most cancer types compared to tissue matched controls (Fig. 3). Aside from KIRP and PRAD for *MMP13*, and KICH and UCEC for *MMP11*, the upregulation was consistent across all cancer types. *MMP11* expression was comparatively high in the normal UCEC tissue, while the other non-significant pairs retained relatively low expression within the cancer samples. Both genes typically featured a very high fold change difference between groups and were among the highest expression differences observed within the dataset (Fig. 1).

### Diagnostic value of MMPs for Cancer

**Fig. 4:**
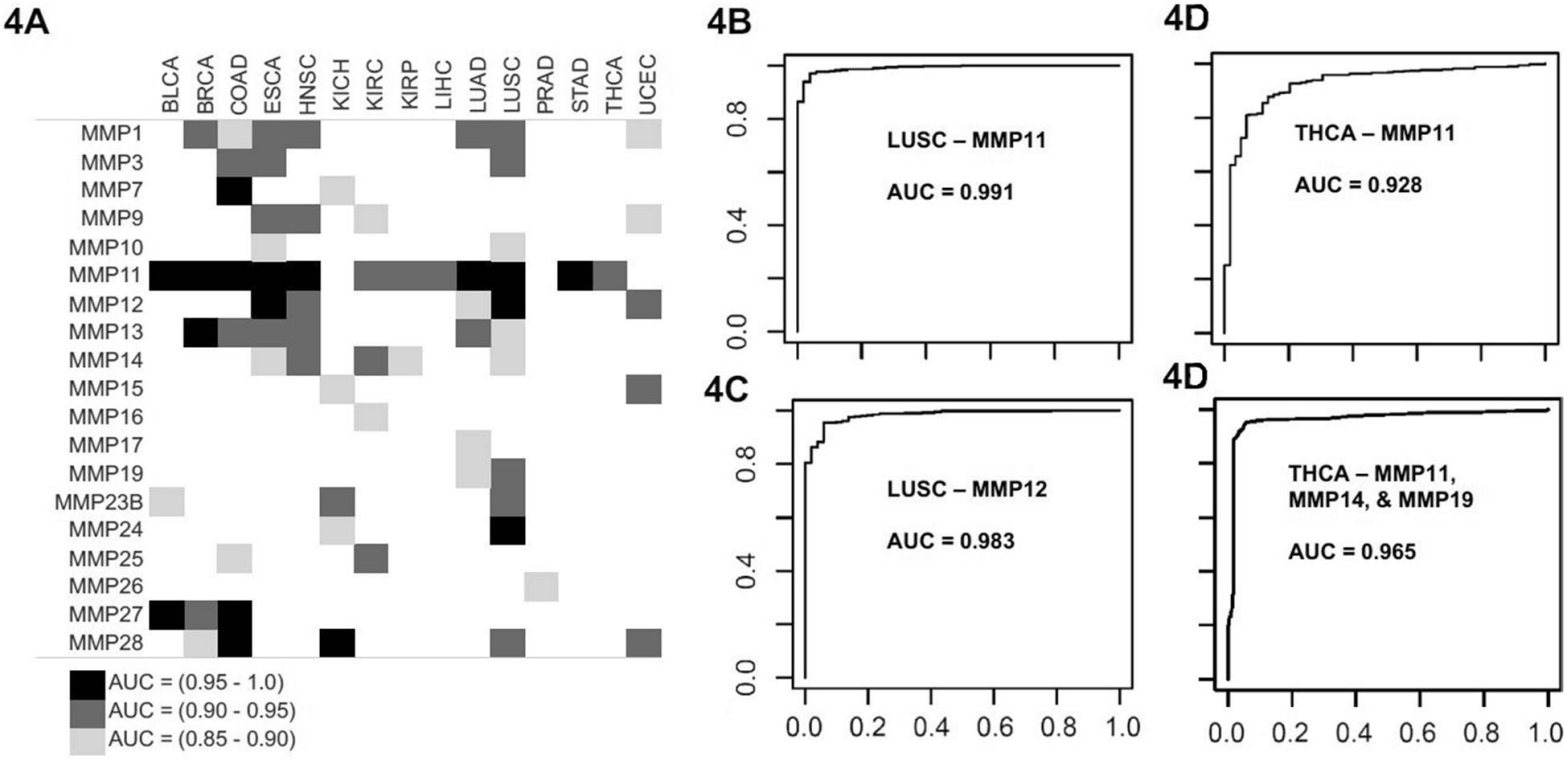
Area under the curve values for receiver operator characteristic (ROC) curves for MMPs across TCGA cancer types. **a** Summary of AUC values. **b** ROC curve for MMP11 in squamous lung cancer. **c** ROC curve for MMP12 in squamous lung cancer. **d** Comparison of univariate analysis of the ROC curve for MMP11 (the MMP with the highest AUC value for thyroid cancer) and multivariate analysis in thyroid cancer combining expression patterns from MMP11, MMP14, and MMP19

The area under the curve (AUC) values for ROC analysis of MMP expression in each cancer are illustrated in Fig. 4a. Values for the range of the AUC as well as the sensitivity values at 90, 95, 99, and 100% specificity are included in Additional file 1: Table S1 for gene-cancer pairs with the highest AUC values. Each cancer type featured at least one MMP with an AUC greater than 0.9 except for PRAD. Six cancer types (BRCA, COAD, ESCA, HNSC, LUSC, and UCEC) showed 4 or more MMPs with AUC greater than 0.9. *MMP11* most frequently had significant predictive values, with AUC greater than 0.9 in twelve cancers and greater than 0.95 in eight. The highest AUC observed for MMP11 was in LUSC with an AUC value of 0.991 (Fig. 4b). Seven MMPs (*MMP7*, *MMP11*, *MMP12*, *MMP13*, *MMP24*, *MMP27*, and *MMP28*) had an AUC of greater than 0.95 for at least one cancer type. For example, *MMP12* had an AUC of 0.983 in lung squamous cancer (LUSC) (Fig. 4b).

### Combinations of dysregulated MMPs strongly predictive of cancer

Using a multivariate analysis, ROC curves were made analyzing expression data using combinations of up to five MMPs for each cancer type. Additional file 1: Figure S1 displays AUC graphs for the top two individual MMPs and combinations of MMPs. The AUC for a combination was usually higher than with a single gene, however was not necessarily superior, as many of the confidence intervals for individual MMPs overlapped with the AUC value of MMPs in combination (Additional file 1: Table S1). For example, for thyroid cancer (THCA), *MMP11* showed an AUC of 0.928 alone, but when combined with *MMP19*, multivariate analysis showed an AUC of 0.961 (Fig. 4c). In colon cancer (COAD), an AUC of 1 was achieved after multivariate analysis of a combination of *MMP7*, *MMP13*, and *MMP28*. The cancer type with the next highest AUC after multivariate analysis was lung squamous (LUSC). Individually, *MMP11* and *MMP12* were the MMPs with the best diagnostic value with an AUC of 0.991 and 0.983 (Fig. 4b), respectively for LUSC. After multivariate analysis two combinations of *MMP11* and *MMP12* yielded an AUC of 0.996.

### Prognostic value of MMPs for Cancer

**Fig. 5:**
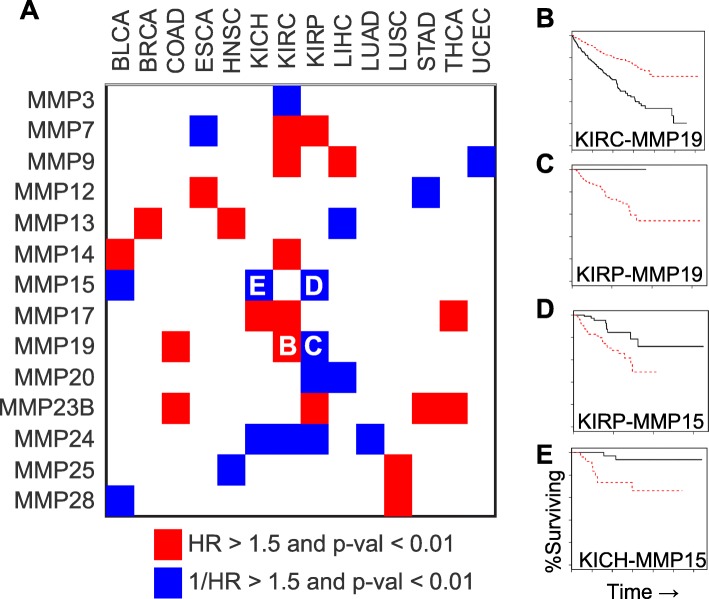
Survival analysis for MMP genes in each TCGA group. **a** Summary of hazard ratios (HR) illustrating cancer-MMP pairs with significant (*p* < 0.01) associations with altered prognosis (HR or 1/HR > 1.5). **b-e** Kaplan Meier plots for **b**) MMP19 in clear cell renal cancer **c**) MMP19 in papillary renal cancer **d**) MMP15 in papillary renal cancer **e**) MMP15 in chromatophobe renal cancer. Hazard ratios, thresholds for expression differences, and *p*-values are shown in Additional file 1: Table S2

Twenty-four proteins from the MMP family were independently analyzed for their ability to predict overall survival in 15 different cancer types using tumor samples from TCGA. Each cancer type was considered independently when examining MMP prediction of overall survival. Hazard ratios, expression thresholds, and *p*-values for the thirty-seven curves that had (*p* < 0.01 with HR or 1/HR > 1.5) are shown in Additional file 1: Table S2 and summarized in Fig. 5a. Of these relationships, sixteen were in a type of renal cell carcinoma (KICH, KIRC, or KIRP). Liver cancer (LIHC) was the only other cancer type with three MMPs associated with a survival change. Genes frequently associated with survival differences included *MMP14*, *MMP17,* and *MMP23B* which were associated with exclusively poorer prognosis for higher expression while *MMP15*, *MMP20*, and *MMP24* were associated exclusively with poorer prognosis for lower expression. Interestingly, several MMPs featured inconsistent survival trends across cancer types with *MMP19* showing opposite trends when comparing renal papillary (KIRP) with renal clear cell (KIRC) (Fig. 5b-c). The strongest hazard ratios were observed for *MMP15* within renal papillary and renal chromophobe (KICH) subtypes (Fig. 5d-e).

## Discussion

### MMP-11 is frequently upregulated in cancer

The observation of *MMP11* upregulation across cancer types is consistent with prior knowledge of gene function facilitating tumor invasion by degrading collagen, fibronectin, and laminin. Upregulated in 13 of 15 cancer types (Fig. 1), *MMP11* expression differences yielded particularly high fold changes and high AUCs. For example, in lung cancers there was a 43-fold increase in squamous (LUSC) and 36-fold increase in adenocarcinoma (LUAD) over control. This upregulation of *MMP11* was contrasted by other MMPs where up-regulation was more cancer-type specific. A reason for this pan-cancer upregulation might be related to the functional ability of MMP-11 in helping evade immune surveillance by desensitizing cancer cells to NK-cells [38]. MMP-11 differs from other MMPs because it is not secreted as a pro-MMP, but is already active upon secretion through intracellular activation by furin, within the constitutive secretory pathway of the trans-Golgi network [39]. Our analysis supports a theory that MMP-11 expression is critical in cancer development and progression.

### MMP-7 is extremely upregulated in colon cancer

MMP-7, a matrilysin protein, had a gene expression fold change of 141.85 in COAD, the highest fold change we found in our analysis. This is consistent with prior studies of colon cancer, that also have indicated that MMP-7 could predict a more aggressive phenotype of colon cancer and correlate negatively with patient survival [40–43]. However, TCGA data did not show any significant associations between survival and *MMP7* expression in colon cancer. MMP-7, a stromelysin, degrades collagens, proteoglycans, elastin, laminin, fibronectin, and casein. It also activates other MMPs, including gelatinase. Upregulation of *MMP7* was primarily found within GI cancers (esophageal, stomach, colon) as well as thyroid and head/neck. Though upregulated in other cancer types, the degree of upregulation for colon cancer, is truly remarkable with a fold change greater than 140. Part of the reason for this level of upregulation could be ascribed to the intrinsic expression of *MMP7* in colon versus other tissues. Additionally, MMP-7 is very closely associated with some of the prime factors in the tumorigenesis of colon cancer. MMP-7 activation is directly associated with APC, and it is well established that APC mutations are frequently implicated as among the first mutations that occurs in the disease history of colon polyps as they progress to cancer [44]. APC mutations often lead to increased β-catenin and thus overexpression of MMP-7. If these changes occur in the earliest stages of colon cancer or precancer as we suspect, MMP-7 may be an excellent early diagnostic marker for colon cancer.

### Downregulation of MMPs

Several MMPs that were largely downregulated in tumor versus control were membrane-type or unclassified by their primary degradation target. MMP-28, also known as epilysin, is downregulated in nine cancer types in our analysis (Fig. 1). This downregulation was highly pronounced in both lung squamous and adenocarcinoma subtypes. The finding is especially intriguing given that stable expression of MMP-28 in lung adenocarcinoma cells results in epithelial mesenchymal transition with accompanying loss of E-cadherin as reported by Illman et al. [37, 45]. Our analysis and prior reports do not capture a clear mechanistic or clinical picture how MMP-28 downregulation interplays with disease progression or metastasis.

### MMPs as diagnostic markers

We believe that MMPs have tremendous potential as diagnostic biomarkers. Survival in cancer can be improved by diagnosis at an early stage. Screening tests such as colonoscopy plus biopsy, the gold standard for colon cancer diagnosis, are often invasive, costly, or uncomfortable while others such as a fecal occult blood test have a lower sensitivity. Identifying novel biomarkers for cancer screening hopefully will lead to highly sensitive, cost-effective, and non-invasive diagnostics. MMPs offer theoretical potential for these properties. Six cancer types featured four or more MMPs with AUC > 0.9 (Fig. 4a). Cancer-by-cancer, lung squamous (LUSC) has two MMPs with sensitivities over 95% (*MMP11* and *MMP12*), colon cancer (COAD) has three over 94% (*MMP11*, *MMP28*, and *MMP7*), and esophageal cancer (ESCA) has two over 94% (*MMP11* and *MMP12*). *MMP11* was 100% specific and over 93% sensitive in colon and esophageal cancer. While, this data indicates that MMPs have the potential to serve as quality biomarkers, confirmation of upregulation in more easily accessed biofluids remains to be determined as increased gene expression does not necessarily mean a protein will be overexpressed at a high enough level to be detected within a biofluid, such as serum, at a high enough level to be acted upon. Will be needed to fulfill the qualities of an excellent, minimally-invasive biomarker. If serum detection is achievable, ubiquitously upregulated MMPs such as *MMP11* or *MMP13* could serve as a pan-cancer marker, capable of identifying the presence of cancer but not the specific location of the cancer.

### MMP combinations as diagnostic markers

Several MMPs interact with each other or rely on other MMPs for their activation. For example, the activation pathway of pro-MMP-2 is cleaved by MMP-14 [2]. It is likely that the dysregulation of one MMP alters the MMP ecosystem and means that MMPs are better predictors when analyzed in combination as opposed to individually. Simultaneous upregulation of MMPs that cleave the same substrates were frequently observed within our data. The combined signal of *MMP11* and *MMP19*, both of which cleave aggrecan and gelatin [46], resulted in a higher AUC value than individually in thyroid cancer. Additionally, stromelysins and collagenases both showed similar patterns of upregulation across tumor types. The inter-dependence and similar functions of MMPs give physiological rationale for performing multivariate analysis when observing MMP dysregulation.

### MMPs as prognostic markers in kidney clear cell carcinoma

MMPs are generally not great prognostic biomarkers for most cancer types. However, MMPs have very good prognostic value for kidney clear cell carcinoma (KIRC) (Fig. 5**)**. High expression of *MMP7*, *MMP9*, *MMP14*, *MMP17* and *MMP19* each individually correlated with a significantly poorer survival in patients with KIRC. Pathologic spread of these tumors from the renal cortex to the renal vein is facilitated by the lack of connective tissue between the renal columns and the vasculature in the renal sinus. Further degradation of the extracellular matrix from higher expression of MMPs could allow invasion of the renal vein and therefore more metastases earlier in the disease course. MMP-2 and -9 are reported in literature to be associated with poor prognosis in kidney clear cell carcinoma [47]. Both MMP-2 and MMP-9 are implicated in angiogenesis which is critical for highly vascularized malignancies such as renal cell carcinoma. Higher expression of MMP-2 and MMP-9 are found in patients who have kidney clear cell carcinoma metastases when compared to patients without distant metastases [48]. Other MMPs, especially those less studied or of lower abundance, may also have critical roles in the promotion of invasion, angiogenesis, and metastasis within this disease state.

The MMP dysregulation in renal cancer might be linked to VHL. VHL mutation, whether inherited or sporadic is frequently implicated in the tumorigenesis of renal clear cell carcinoma. With the loss of VHL, HIF is no longer properly degraded and free to induce the expression of proteins needed in hypoxic conditions. This leads to the upregulation of *MMP14* in kidney clear cell carcinoma in patients with deletion of VHL [49]. We found that patients with higher expression of *MMP14* had generally poorer survival, consistent with the more aggressive and invasive malignancy associated with VHL functional loss. Additionally, VHL regulates the assembly of collagen IV, a major constitute of the basement membrane and target of MMP-3, MMP-8, and MMP-9 [50]. This profile likely results in increased destruction and invasion through the basement membrane and may explain why higher expression of MMP-3 correlates with a poorer prognosis in kidney clear cell carcinoma patients [51]. Our evidence corroborates the literature knowledge of the significant role of MMPs in the disease history of kidney cancer.

## Conclusions

This study had a number of strengths, including that it is the largest MMP gene expression analysis to date. It encompassed all MMP genes using RNA-seq technology across 15 different types of cancers. This manuscript explored both the significance of MMP expression in comparison to normal tissue expression and observed how MMP levels associate with survival differences. However, this manuscript had some limitations as it relates to the TCGA. One limitation was that based on information from the TCGA, it was not feasible for us to correlate how a tumor’s stroma and supporting cells contribute to MMP expression. Other limitations include that we did not perform any analyses which correalated MMP expression to demographic or pathologic factors outside of tumor histological subtype. Despite these limitations, the findings within this manuscript are still relevant.

The information gained from this analysis support that several MMPs are almost ubiquitously upregulated across different cancer types while others are more specific to certain cancers. Based on the information presented in this manuscript, we believe the most actionable point is an investigation into the minimally invasive diagnostic capabilities of the MMPs which were the most ubiquitously upregulated across cancers such as *MMP11* and *MMP13*. Functional studies or further exploration of these genes as potential pan-cancer biomarkers may provide a sizeable benefit to the medical community.

## Additional file

Additional file 1: **Figure S1**. Top univariate and multivariate ROC curves for MMP-cancer pairs. **Table S1**. Sensitivity values for high AUC MMP-cancer pairs. **Table S2**. Significant survival differences associated with altered MMP expression. (PDF 1499 kb)

## Data Availability

Raw data was obtained from TCGA repositories and were obtained for our purposes from (https://xenabrowser.net/datapages/). Analyzed data is available within supplementary information or from the authors upon reasonable request.

## Abbreviations

BLCA: Bladder Cancer
BRCA: Breast Cancer
COAD: Colon Adenocarcinmoa
ECM: Extracellular Matrix
EMT: epithelial mesenchymal transition
ESCA: Esophageal Cancer
FC: Fold Change
HIF: Hypoxia Induced Factor
HNSC: Head and Neck Cancer
HR: Hazard Ratio
KICH: Kidney Chromophobe
KIRC: Renal Clear Cell Cancer
KIRP: Renal Papillary Cancer
LIHC: Hepatocellular Cancer
LUAD: Lung Adenocarcinoma
LUSC: Lung Squamous Cancer
MMP: Matrix Metalloproteases
PRAD: Prostate Adenocarcinoma
pval: *p*-value
ROC: Receiver Operator Characteristic
STAD: Stomach Adenocarcinoma
TCGA: The Cancer Genome Atlas
THCA: Thyroid Cancer
UCEC: Uterine Corpus Endometrial Carcinoma
VHL: Von Hippel-Lindau
