# Supplementary material for: A pan-cancer perspective of matrix metalloproteases (MMP) gene expression profile and their diagnostic/prognostic potential
Source: BMC Cancer. 2019 Jun 14;19:581. doi: 10.1186/s12885-019-5768-0 (PMC6567474; doi:10.1186/s12885-019-5768-0)
Supplement: Supplementary file 1 — Figure S1. Top univariate and multivariate ROC curves for MMP-cancer pairs. Table S1. Sensitivity values for high AUC MMP-cancer pairs. Table S2. Significant survival differences associated with altered MMP expression. (PDF 1499 kb) [file 12885_2019_5768_MOESM1_ESM.pdf]

Supp. Table 1

Cancer	Protein	AUC	90	95	99	100							
BLCA	MMP27	0.96 (0.93 - 0.99)	94.7	89.5	63.2	31.6	KIRC	MMP25	0.934 (0.924 - 0.944)	85.7	80.5	54	32.5
	MMP11	0.949 (0.936 - 0.962)	92.6	91.7	73.2	73.2		MMP14	0.922 (0.911 - 0.933)	83.5	71.1	40.7	30.4
	MMP23B	0.881 (0.832 - 0.93)	63.2	42.1	15.8	0		MMP11	0.916 (0.904 - 0.928)	79.9	64	25.5	21.6
BRCA	MMP11	0.994 (0.992 - 0.996)	98.7	98.2	92.3	85.3		MMP9	0.882 (0.867 - 0.897)	63	57	43.9	41.1
	MMP13	0.954 (0.948 - 0.96)	90.3	77.6	61.9	55.7		MMP16	0.863 (0.846 - 0.88)	66.8	54	51.6	49.9
	MMP1	0.931 (0.923 - 0.939)	74.3	68.9	55.6	51.4	KIRP	MMP11	0.921 (0.905 - 0.937)	76.9	70	37.6	37.6
	MMP27	0.904 (0.886 - 0.922)	73.7	64	36.8	0		MMP14	0.901 (0.882 - 0.92)	77.6	68.6	59.3	59.3
	MMP28	0.855 (0.833 - 0.877)	60.5	52.6	30.7	0	LIHC	MMP11	0.916 (0.902 - 0.93)	81.7	78.2	64.7	62
COAD	MMP11	0.989 (0.984 - 0.994)	97.9	97.2	96.2	96.2	LUAD	MMP11	0.977 (0.972 - 0.982)	97.5	90.1	78.6	35.9
	MMP7	0.983 (0.977 - 0.989)	97.2	94.8	87.1	87.1		MMP13	0.912 (0.899 - 0.925)	70.3	63.5	55.2	51.3
	MMP28	0.98 (0.965 - 0.995)	100	92.7	41.5	0		MMP1	0.909 (0.896 - 0.922)	75.3	62.9	55.5	52
	MMP27	0.957 (0.936 - 0.978)	90.2	87.8	82.9	0		MMP12	0.888 (0.872 - 0.904)	72.8	57.5	42.5	38.5
	MMP13	0.927 (0.912 - 0.942)	79.4	78.3	37.8	37.8		MMP19	0.878 (0.85 - 0.906)	57.6	40.7	22	5.08
	MMP3	0.907 (0.89 - 0.924)	75.9	60.8	50.4	50.4		MMP17	0.858 (0.84 - 0.876)	73.6	72.2	69.1	67.4
	MMP1	0.899 (0.881 - 0.917)	79.4	75.5	42.3	42.3	LUSC	MMP11	0.991 (0.988 - 0.994)	97.8	97.6	93.8	86.5
	MMP25	0.865 (0.829 - 0.901)	58.5	41.5	2.44	0		MMP12	0.983 (0.978 - 0.988)	95.6	95.4	86.3	80.5
ESCA	MMP12	0.984 (0.976 - 0.992)	97.3	97.3	86.4	86.4		MMP24	0.975 (0.96 - 0.99)	94.1	90.2	35.3	1.96
	MMP11	0.979 (0.969 - 0.989)	94	94	93.5	93.5		MMP1	0.947 (0.937 - 0.957)	84.5	81.7	74.7	66.3
	MMP3	0.938 (0.917 - 0.959)	92.4	92.4	73.4	73.4		MMP28	0.944 (0.922 - 0.966)	92.2	52.9	0	0
	MMP9	0.926 (0.902 - 0.95)	87	87	71.2	71.2		MMP19	0.931 (0.907 - 0.955)	76.5	56.9	31.4	0
	MMP1	0.92 (0.895 - 0.945)	88	88	51.1	51.1		MMP3	0.924 (0.912 - 0.936)	81.3	76.9	71.9	70.7
	MMP13	0.916 (0.89 - 0.942)	84.2	84.2	48.4	48.4		MMP23B	0.905 (0.878 - 0.932)	72.6	41.2	7.84	0
	MMP10	0.898 (0.867 - 0.929)	90.2	90.2	35.9	35.9		MMP10	0.896 (0.88 - 0.912)	78.5	71.9	65.9	60.6
	MMP14	0.898 (0.867 - 0.929)	83.2	83.2	39.7	39.7		MMP14	0.894 (0.878 - 0.91)	79.5	71.7	55	19.5
HNSC	MMP11	0.964 (0.957 - 0.971)	84.2	83.7	69.4	69.4		MMP13	0.886 (0.869 - 0.903)	77.7	74.7	58.2	52
	MMP9	0.941 (0.93 - 0.952)	80.2	68.1	58.3	58.3	PRAD	MMP26	0.863 (0.844 - 0.882)	68	52.1	33.4	25.8
	MMP12	0.933 (0.921 - 0.945)	76.9	60.6	49.8	49.8	STAD	MMP11	0.964 (0.956 - 0.972)	92.8	87.2	81.7	81.7
	MMP14	0.918 (0.904 - 0.932)	78.5	76.2	11.7	11.7	THCA	MMP11	0.928 (0.917 - 0.939)	81.6	72.5	62.4	25.2
	MMP1	0.913 (0.899 - 0.927)	74	63.3	5	5	UCEC	MMP28	0.933 (0.899 - 0.967)	79.2	62.5	54.2	25
	MMP13	0.913 (0.899 - 0.927)	78.3	50.8	44.4	44.4		MMP15	0.912 (0.891 - 0.933)	81.8	77.3	68.8	68.8
KICH	MMP28	0.962 (0.936 - 0.988)	96	68	56	8		MMP12	0.904 (0.881 - 0.927)	71	59.1	59.1	59.1
	MMP23B	0.94 (0.907 - 0.973)	72	64	24	20		MMP1	0.876 (0.849 - 0.903)	59.7	18.8	1.14	1.14
	MMP24	0.887 (0.854 - 0.92)	75.8	75.8	74.2	74.2		MMP9	0.872 (0.844 - 0.9)	68.8	58	51.7	51.7
	MMP7	0.884 (0.84 - 0.928)	56	48	16	4							
	MMP15	0.875 (0.841 - 0.909)	80.3	68.2	62.1	62.1							

Supp. Fig 1

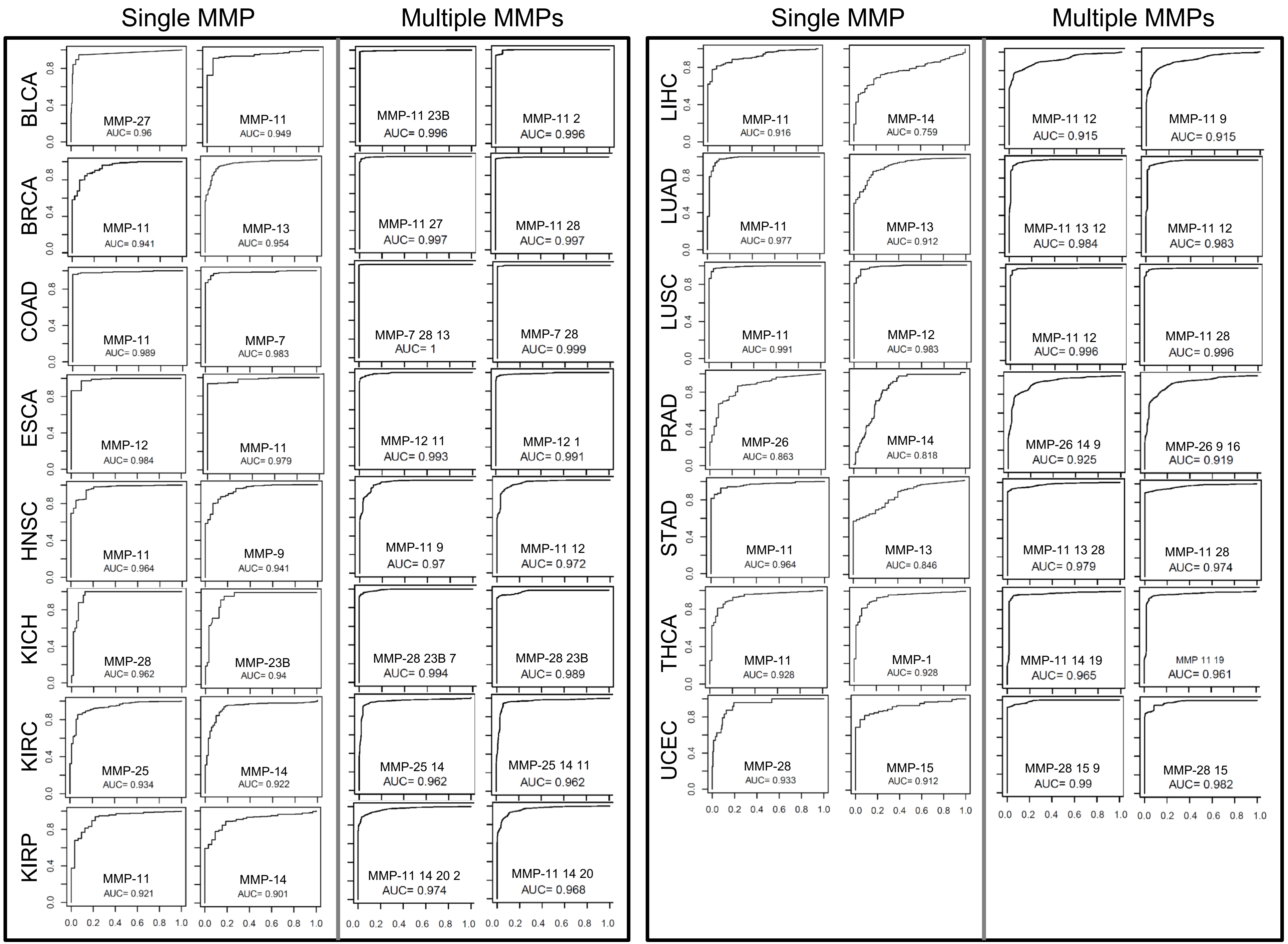
Single MMP
Multiple MMPs
Single MMP
Multiple MMPs
BLCA
MMP-27
AUC= 0.96
MMP-11
AUC= 0.949
MMP-11 23B
AUC= 0.996
MMP-11 2
AUC= 0.996
BRCA
MMP-11
AUC= 0.941
MMP-13
AUC= 0.954
MMP-11 27
AUC= 0.997
MMP-11 28
AUC= 0.997
COAD
MMP-11
AUC= 0.989
MMP-7
AUC= 0.983
MMP-7 28 13
AUC= 1
MMP-7 28
AUC= 0.999
ESCA
MMP-12
AUC= 0.984
MMP-11
AUC= 0.979
MMP-12 11
AUC= 0.993
MMP-12 1
AUC= 0.991
HNSC
MMP-11
AUC= 0.964
MMP-9
AUC= 0.941
MMP-11 9
AUC= 0.97
MMP-11 12
AUC= 0.972
KICH
MMP-28
AUC= 0.962
MMP-23B
AUC= 0.94
MMP-28 23B 7
AUC= 0.994
MMP-28 23B
AUC= 0.989
KIRC
MMP-25
AUC= 0.934
MMP-14
AUC= 0.922
MMP-25 14
AUC= 0.962
MMP-25 14 11
AUC= 0.962
KIRP
MMP-11
AUC= 0.921
MMP-14
AUC= 0.901
MMP-11 14 20 2
AUC= 0.974
MMP-11 14 20
AUC= 0.968
LIHC
MMP-11
AUC= 0.916
MMP-14
AUC= 0.759
MMP-11 12
AUC= 0.915
MMP-11 9
AUC= 0.915
LUAD
MMP-11
AUC= 0.977
MMP-13
AUC= 0.912
MMP-11 13 12
AUC= 0.984
MMP-11 12
AUC= 0.983
LUSC
MMP-11
AUC= 0.991
MMP-12
AUC= 0.983
MMP-11 12
AUC= 0.996
MMP-11 28
AUC= 0.996
PRAD
MMP-26
AUC= 0.863
MMP-14
AUC= 0.818
MMP-26 14 9
AUC= 0.925
MMP-26 9 16
AUC= 0.919
STAD
MMP-11
AUC= 0.964
MMP-13
AUC= 0.846
MMP-11 13 28
AUC= 0.979
MMP-11 28
AUC= 0.974
THCA
MMP-11
AUC= 0.928
MMP-1
AUC= 0.928
MMP-11 14 19
AUC= 0.965
MMP 11 19
AUC= 0.961
UCEC
MMP-28
AUC= 0.933
MMP-15
AUC= 0.912
MMP-28 15 9
AUC= 0.99
MMP-28 15
AUC= 0.982
0.0
0.4
0.8
0.0 0.2 0.4 0.6 0.8 1.0

Supplementary Table 2: Significant survival differences associated with altered MMP expression

Cancer	Gene	Cutoff Percentile	HR	1/HR	p-val
KICH	MMP15	60	<0.1	>20	1.6E-03
KIRP	MMP15	90	<0.1	>20	9.8E-04
KIRP	MMP19	90	<0.1	>20	3.3E-03
KIRP	MMP24	90	<0.1	>20	6.1E-03
KICH	MMP24	10	<0.1	10.6	2.0E-03
KICH	MMP17	40	6.0	0.2	5.3E-03
THCA	MMP23B	90	4.4	0.2	7.7E-03
ESCA	MMP7	90	0.3	4.0	2.9E-03
UCEC	MMP9	10	0.3	3.5	7.3E-03
THCA	MMP17	70	3.3	0.3	8.4E-03
KIRP	MMP23B	90	3.0	0.3	9.1E-03
KIRC	MMP19	20	2.7	0.4	4.4E-06
KIRP	MMP20	10	0.4	2.7	6.7E-04
KIRP	MMP7	80	2.6	0.4	5.3E-03
LUAD	MMP24	10	0.4	2.4	5.5E-05
COAD	MMP19	30	2.4	0.4	2.4E-03
KIRC	MMP14	90	2.2	0.5	4.8E-04
KIRC	MMP9	40	1.5	0.7	8.1E-03
KIRC	MMP24	10	0.5	2.0	2.2E-03
ESCA	MMP12	40	2.0	0.5	3.9E-03
COAD	MMP23B	70	1.9	0.5	8.5E-03
BRCA	MMP13	90	1.9	0.5	4.2E-03
BLCA	MMP14	20	1.9	0.5	2.7E-03
HNSC	MMP13	20	1.9	0.5	1.2E-03
STAD	MMP23B	40	1.8	0.5	3.7E-04
KIRC	MMP17	20	1.8	0.6	1.1E-03
LIHC	MMP20	20	0.6	1.8	1.5E-03
HNSC	MMP25	10	0.6	1.7	9.5E-03
LIHC	MMP9	40	1.7	0.6	5.2E-03
BLCA	MMP15	40	0.6	1.7	8.0E-04
KIRC	MMP3	60	0.7	1.4	2.8E-04
BLCA	MMP28	70	0.6	1.6	5.0E-03
KIRC	MMP7	40	1.6	0.6	2.9E-03
LIHC	MMP13	70	0.8	1.3	8.4E-03
LUSC	MMP28	60	1.6	0.6	1.6E-03
LUSC	MMP25	70	1.5	0.6	3.9E-03
STAD	MMP12	60	0.6	1.5	8.2E-03
